# From bench to bedside: advancing our understanding of radioresistance in rectal cancer

**DOI:** 10.1007/s10555-026-10359-6

**Published:** 2026-07-22

**Authors:** Diogo Estêvão, Miguel da Cruz-Ribeiro, Diogo Coelho, Joana Lencart, Olga Sousa, Bruno Sarmento, Olivier de Wever, Maria J. Oliveira, Tânia Cruz

**Affiliations:** 1 i3S - Institute for Research and Innovation in Health, University of Porto, Rua Alfredo Allen, 208, 4200-135 Porto, Portugal; 2 ICBAS – School of Medicine and Biomedical Sciences, University of Porto, Porto, Portugal; 3 Laboratory of Experimental Cancer Research, Department of Human Structure and Repair, Ghent University, Ghent, Belgium; 4 IUCS – University Institute of Health Sciences (CESPU), Gandra, Portugal; 5 Medical Physics, Radiobiology and Radiation Protection Group, IPO Porto Research Center (CI-IPOP), Portuguese Oncology Institute of Porto (IPO Porto), Porto, Portugal; 6 Medical Physics Department, Portuguese Oncology Institute, Porto, Portugal; 7 Department of Radiotherapy, Portuguese Oncology Institute of Porto (IPO Porto), Porto, Portugal

**Keywords:** Rectal cancer, Radioresistance, Radiobiology Rs, Metabolic rewiring

## Abstract

The global incidence of rectal cancer (RC) is increasing at an alarming rate, with over 50% of cases still diagnosed at advanced stages, despite improvements in early detection. While neoadjuvant (chemo)radiotherapy regimens remain a cornerstone of treatment, up to 20–40% of tumours exhibit or develop resistance to ionising radiation, limiting therapeutic options and leading to disease evolution. This review offers a comprehensive insight into the complex biological and molecular mechanisms underlying RC radioresistance within the fundamental 6Rs of radiobiology: Repair, Reoxygenation, Redistribution, Radiosensitivity, Repopulation, and Reactivation. More specifically, DNA damage repair pathways, cell cycle regulation, hypoxia, transcriptional plasticity regulators such as cancer stem cells, and non-coding RNAs are comprehensively reviewed. Additionally, the critical roles of tumour-infiltrating lymphocytes, cancer-associated macrophages, and inflammatory cancer-associated fibroblasts in radioresistance are also dissected, which collectively shape an immunosuppressive and pro-metastatic niche following radiotherapy. Furthermore, a less explored mechanism, namely the metabolic rewiring of cancer cells after radiotherapy, is proposed as a new 7th R of Radiobiology, namely “Reprogramming”, that enables tumour survival and promotes aggressive phenotypes. This new perspective adds a new layer of complexity to the molecular understanding of RC resistance and provides a mechanistic insight currently missing in radiobiology research. Additionally, advanced experimental models, such as spheroids, patient-derived organoids, and animal models, are discussed as valuable platforms for pre-clinical research and therapeutic testing. This review also integrates mechanistic insights with biomarker-guided clinical decision-making to support RC management. By unravelling these multifactorial mechanisms, we highlight opportunities to develop predictive biomarkers and tailored therapeutic strategies to overcome resistance and improve patient outcomes.

## Introduction: the growing challenge of RC radioresistance

Rectal cancer (RC) is the eighth most incident malignancy worldwide and the tenth leading cause of cancer-related deaths, accounting for approximately 30–40% of all colorectal cancer cases [1]. Of particular concern is the increasing incidence of RC in young adults under 35 years of age, who frequently present more advanced and aggressive disease stages [2]. This rising trend has been associated with modifiable lifestyle habits, including high consumption of red and processed meats and insufficient physical activity, along with alterations in the gut microbiome [3, 4]. Interestingly, a recent study reported that the colibactin-associated mutational signatures SBS88 and ID18, which are linked to toxin-producing strains of *Escherichia coli* and other *Enterobacteriaceae* within the gut microbiota, were 2.5 and 4 fold more frequent, respectively, in colorectal cancers diagnosed before the age of 50 [5]. Additionally, an increased abundance of *Fusobacterium* and *Flavonifractor* genera has been identified in younger rectal cancer patients [6]. Furthermore, genetic predisposition syndromes such as Lynch syndrome or familial adenomatous polyposis, as well as increased mutation rates at codon 279 of the *APC* gene, also contribute to early-onset CRC [7, 8]. Also, environmental exposures, including the herbicide picloram, have been implicated in the development of early-onset colon and rectal cancer [9].

Overall, approximately 60–70% of rectal tumours are diagnosed at more advanced stages (II and III), requiring a multidisciplinary treatment approach that usually includes neoadjuvant radiotherapy alone or in combination with chemotherapy, followed by total mesorectal excision [10]. Recent phase III clinical trials demonstrated that enhanced neoadjuvant strategies, specifically by combining preoperative radiotherapy with more potent chemotherapy regimens, improve disease outcomes, showing a significant increase in pathological complete response (pCR) rates, as well as improvements in patientsʼ disease-free (DFS) and overall survival (OS) [11, 12]. These increased pCR rates with multimodal chemoradiotherapy regimens have reinforced interest in organ-preserving approaches, leading to the implementation of watch-and-wait strategies, in which an estimated 50% of patients may avoid surgery without compromising oncologic safety and, consequently, increasing quality of life [13, 14]. Nowadays, the most widely adopted neoadjuvant regimens include either short-course (25 Gy in 5 fractions over 5 consecutive days) or long-course (45–50 Gy in 25–28 daily fractions) radiotherapy, both typically combined with radiosensitising chemotherapy. This is usually followed by total mesorectal excision and, in many cases, adjuvant chemotherapy [15–17]. Although these schemes are still the most frequently used in the clinic, there has been a shift towards a total neoadjuvant approach (TNT), in which both systemic chemotherapy and chemoradiotherapy are administered in the neoadjuvant setting, increasing pCR rates [11, 12, 18]. Nevertheless, despite these multimodal approaches, approximately 20–40% of RC patients still experience loco-regional recurrence, which can ultimately progress to metastatic disease [19, 20]. These observations underscore that radioresistance is not merely a barrier to local tumour control, but a critical determinant of tumour evolution and systemic dissemination.

Given the substantial rate of RC recurrence, the increasing incidence of this disease, especially among young individuals, and the pivotal role of radiotherapy in current treatment protocols, it is essential to elucidate the biological and molecular mechanisms underlying radioresistance. Although still incompletely characterised, radioresistance mechanisms in RC have been associated with several tumour-intrinsic (e.g., anatomic location, size, and angiogenic profile) and patient-related (e.g., overall clinical status, genetic defects, and immune profile) factors that may be present across multiple radiotherapy-treated tumours [21]. In this review, an updated overview of the main biological radioresistance factors in RC, with particular focus on regulatory genes and proteins, metabolic reprogramming, the transcriptional plasticity players, and the modulatory effects of the tumour microenvironment, namely the tumour-infiltrating lymphocytes (TILs), tumour-associated macrophages (TAMs), and cancer-associated fibroblasts (CAFs), will be addressed. Finally, a prospective summary will outline emerging experimental models and research tools expected to play a pivotal role in advancing mechanistic understanding, as well as the translational implications for biomarker research in the context of RC radiotherapy.

## Unravelling the biological mechanisms of RC radioresistance

The fundamental principle behind radiotherapy, including the fractionated schemes commonly used in clinical practice, is to induce DNA damage [22]. Ionising radiation can lead to a variety of DNA alterations, including oxidised bases, abasic sites, single-strand DNA breaks, and most critically, double-strand DNA breaks (DSBs), which are the most cytotoxic form of DNA damage [23]. Cancer cells often exhibit deficiencies in their DNA damage response machinery, making them less efficient at repairing DSBs than healthy cells. This repair inefficiency results in the accumulation of DNA errors, consequently triggering cell death.

In 1975, Withers introduced the four principles of radiotherapy, namely Repair, Reoxygenation, Redistribution, and Repopulation, which are fundamental to understanding the therapeutic effects of fractionated radiotherapy [24]. Repair refers to the greater capacity of healthy cells to repair DNA damage compared to cancer cells under fractionated doses of ionising radiation [23]. Reoxygenation refers to the increase in oxygen availability within previously hypoxic tumour regions that occurs after fractionation radiotherapy, resulting from tumour shrinkage, thereby enhancing the production of reactive oxygen species (ROS) through radiolysis, leading to DNA damage and radiosensitivity [25]. Additionally, one of the main advantages of fractionated radiotherapy is its ability to allow more resistant cells to progress into phases of the cell cycle that are more sensitive to ionising radiation, such as the G_2_-M phase, thereby enhancing treatment effectiveness. This process is referred to as Redistribution [26]. Indeed, cells synchronised in G_2_ and M phases are more susceptible to apoptosis than those in G_1_ or S phase. This increased susceptibility is mainly due to enhanced DNA repair mechanisms active during S phase, which are integral to DNA replication. Cells in the S phase exhibit higher levels of the antioxidant glutathione, providing increased protection against ROS and, consequently, reducing the likelihood of cell death [27]. Repopulation, the fourth principle, refers to the proliferation of surviving clonogenic cells during treatment, leading to the regeneration of healthy tissue after radiotherapy-induced cell death [28]. However, it was not until 14 years later that Steel and colleagues introduced Radiosensitivity as a fifth principle, exploring ionising radiation sensitivity across different tissues and cell types [29]. These variations help explain differences in treatment protocols across multiple tumours. For example, glioblastoma, one of the most radioresistant tumours, is currently treated with a daily ionising radiation fraction of 2 Gy over 30 days, reaching a total of 60 Gy, whereas advanced Hodgkin lymphoma is treated with 30–36 Gy of ionising radiation delivered in 15–20 fractions [30, 31]. More recently, a sixth principle, known as Reactivation, has been proposed by Boustani and colleagues [32]. The concept explores the activation of the immune system by the release of tumour-associated antigens resulting from radiotherapy-induced cell death. These antigens are subsequently recognised, processed and presented by the antigen-presenting cells, which in turn migrate to proximal lymph nodes to stimulate effector T cells and trigger an immune response [33].

Overall, the effectiveness of fractionated radiotherapy, driven by these six interrelated principles, has significantly improved OS and DFS in most tumours. Nevertheless, in some cases, tumour cells counteract ionising radiation effects by exploiting different biological mechanisms, leading to treatment failure, tumour recurrence and ultimately to metastatic spread.

RC is particularly prone to radioresistance, a phenomenon linked to transcriptomic and proteomic dysregulation alongside metabolic reprogramming, proposed in this manuscript as an emerging seventh R of radiobiology. While the concept of metabolic reprogramming has been recognised, especially in glioblastoma and pancreatic cancers, its formal acceptance as a radiobiology principle specific to RC has, to our knowledge, not been previously proposed [34–38]. This conceptual extension of the already established 6Rs of radiobiology is supported by growing evidence linking lipid metabolism, iron homeostasis, and one-carbon metabolism to ionising radiation exposure in CRC models, as reviewed herein.

### Repair

Radiotherapy induces DNA damage in cancer cells, primarily through the generation of DNA DSBs, which are among the most lethal forms of DNA lesions. If not properly repaired, these breaks can lead to mitotic catastrophe and, consequently, cell death. Because cancer cells often exhibit deficiencies in DNA repair mechanisms, they are more susceptible to ionising radiation-induced damage than healthy cells. This selective vulnerability highlights one of radiotherapyʼs therapeutic windows [23].

**Fig. 1:**
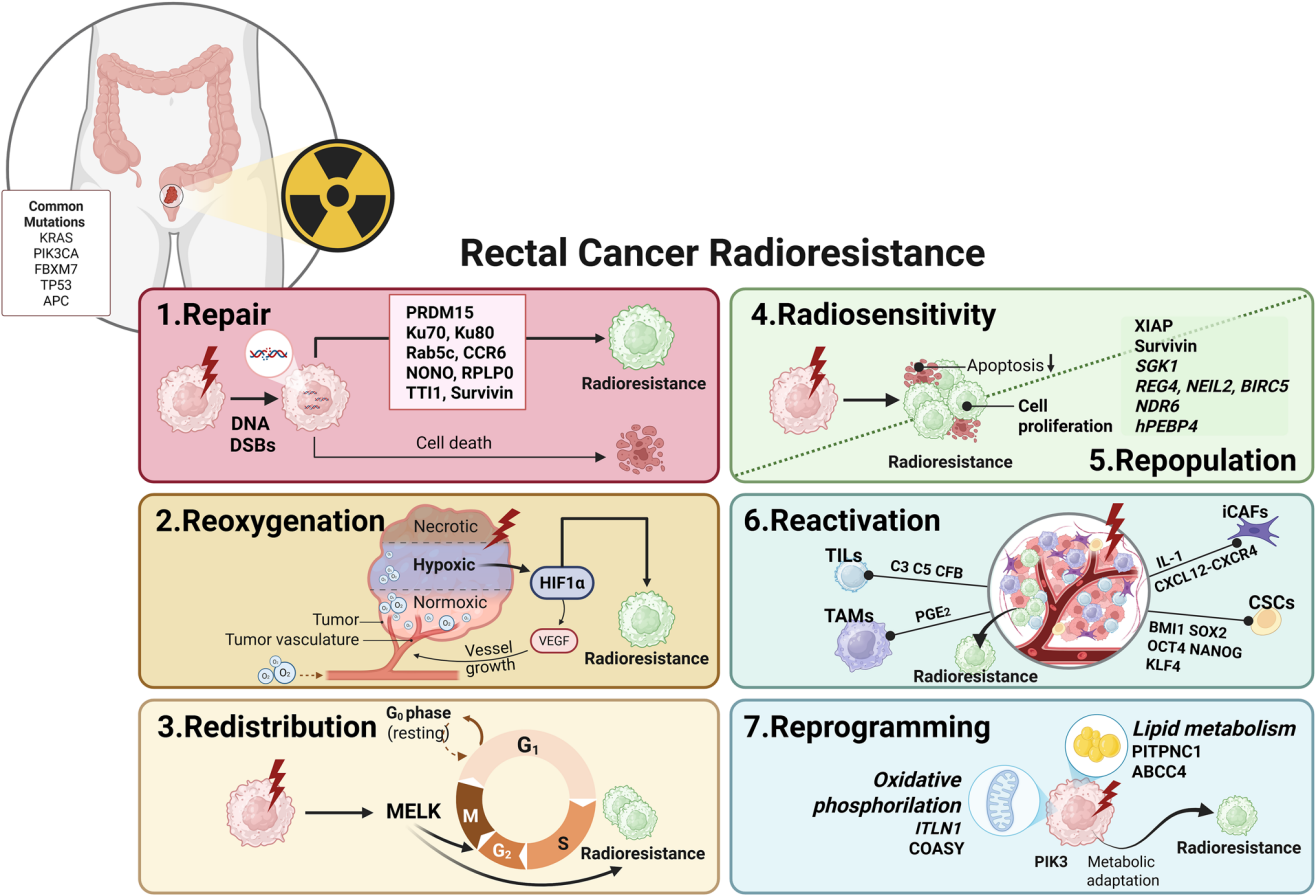
Mechanisms of RC radioresistance, the 7Rs of radiobiology. RC radioresistance is a multifactorial process that subverts radiobiological principles. Despite the genetic mutations found in RC cells, multiple proteins and genes are altered to prevent ionising radiation-mediated cell death. Among these, DNA repair players such as PRDM15, Ku70, Ku80, Rab5c, CCR6, NONO, RPLP0, and TTI1 have been shown to contribute to cell survival. Hypoxia and HIF-1α have also been identified as key to RC radioresistance, also supported by MELK-mediated cell-cycle redistribution, arresting cells in more radioresistant phases [41]. The latter aligns with the repopulation of the tumour; once submitted to radiotherapy, it deploys XIAP, Survivin, SGK1, REG4, NEIL2, BIRC5, and hPEBP4 to promote cancer cell proliferation and minimise apoptosis. Additionally, modulation of the TME immune compartment toward a more immunosuppressive milieu promotes cancer cell escape and survival. Lastly, the reprogramming of metabolic pathways, including oxidative phosphorylation and lipid metabolism via ITLN1, COASY, PITPNC1, and ABCC4, results in tumour adaptation, supporting RC radioresistance

The DNA damage response (DDR) is a complex network of signalling pathways activated to detect and repair DSBs. In fractionated radiotherapy schemes, the timing between ionising radiation doses allows healthy tissue to recover, due to more robust DNA repair mechanisms, while tumour cells accumulate lethal damage. However, RC cells often exhibit alterations in key DDR components that confer a survival advantage and contribute to resistance to radiotherapy-induced cell death (Fig. 1). Yu Zu and colleagues have shown that PR/SET Domain 15 (PRDM15), a DNA-binding transcription regulator involved in the reprogramming of pluripotent stem cells and on cell fate decisions, plays a key role in conferring radioresistance to RC cells. PRDM15 interacts with the DNA-dependent protein kinase (DNA-PKcs*)* and with Ku70 and Ku80 heterodimers. It facilitates DNA damage repair, such as the Non-homologous end-joining (NHEJ) mechanism, by recruiting nucleases, polymerases, and ligases to add novel nucleotides to the broken ends and join them together [39, 40]. In fact, PRDM15 expression is increased in tissues from RC patients who are resistant to radiotherapy, and it is associated with poor prognosis. Additionally, *in vitro* studies have shown that PRDM15 co-localises with *γ-H2AX* foci, a histone that is rapidly up-regulated in response to DSBs, immediately interacting with DNA repair machinery. Moreover, downregulation of *PRDM15* expression in 2D colon cancer cell lines led to reduced cell proliferation, lower survival rates after exposure to 2, 5, and 6 Gy of ionising radiation, decreased accumulation of cells at the G_2_/M checkpoint, and consequently increased cell death. In line with these findings, tumours derived from *PRDM15*-knockdown HCT116 cells exposed to 15 Gy showed increased necrosis, enhanced cell death, and reduced proliferative capacity compared with *PRDM15*-negative controls [40].

Another key study identified increased levels of Ku70, Ku80, and Rab5c, transport proteins commonly involved in NHEJ DNA repair, in residual tumours from patients who relapse after neoadjuvant chemoradiotherapy (nCRT). This was further supported by the increased expression of Ku70, Ku80, and Rab5c observed in the rectal adenocarcinoma cell line SW837 after being subjected to 6 Gy of ionising radiation, consequently leading to the internalisation of the Epidermal Growth Factor Receptor (EGFR), mediated by the Rab5c trafficking protein. Given that previous research has shown that nuclear EGFR modulates the expression of DNA repair proteins, such as Ku70 and Ku80*,* it is suggested that SW837 RC cells exposure to ionising radiation further enhances the activation of these DNA repair proteins, promoting a more efficient DNA damage repair response compared to parental cells [42, 43] (Fig. 1).

In line with these findings, CCR6, a G protein-coupled receptor for CCL20 that guides the recruitment and migration of immune cells, was found to be overexpressed in radioresistant RC patients compared with those who achieved pCR (76.3% vs 37.5%, respectively) [44]. Interestingly, *in vitro* results revealed that *CCR6* suppression increased the radiosensitivity of LoVo colon cancer cells. However, *CCR6* silencing did not directly induce DNA damage, but rather impaired DNA damage repair efficiency, as indicated by the higher percentage of γ-H2AX-positive cells observed in transgenic LoVo cells, 4 h after exposure to 2 Gy. Although the exact cellular mechanism by which CCR6 impairs the response to radiotherapy-induced DNA damage was not fully explored, the authors hypothesise that this may occur through activation of Akt and ERK signalling pathways. In addition, the CCR6-CCL20 axis appears to promote the recruitment and upregulation of key NHEJ proteins, such as Ku70 and DNA-PKcs, which are primarily responsible for repairing radiotherapy-induced DSBs during the G_1_ phase of the cell cycle [44–47] (Fig. 1). Another study led by Wang and colleagues identified the DNA/RNA-binding Non-POU domain-containing octamer-binding (NONO) protein, together with the ribonucleoprotein lateral stalk subunit P0 (RPLP0), as modulators of RC radioresistance. These two proteins appear to work together to enhance DNA-PK activation during NHEJ-mediated DSB repair by promoting autophosphorylation at Thr2609 [48]. By evaluating the clinical significance of the NONO and RPLP0 protein complex in biopsies of patients with locally advanced rectal cancer (LARC), the authors observed that this complex was highly expressed in patients with Tumour Regression Grade (TRG) 3 (> 50% residual carcinoma present) after chemoradiotherapy treatment. Indeed, *in vitro* studies showed that RPLP0 is a novel NONO-binding protein, significantly upregulated in HCT116 colon cancer cells following 5 Gy of ionising radiation. Silencing of *RPLP0* resulted in reduced levels of γ-H2AX and fewer radiation-induced γ-H2AX foci, suggesting impaired DNA DSB repair. This reduction in γ-H2AX foci was partially reversed by RPLP0 overexpression [48] (Fig. 1). Recently, TELO2-interacting protein 1 (TTI1), a DNA damage response molecule, was shown to play a crucial role in activating DNA damage repair mechanisms by assembling and stabilising the ATM protein, a serine/threonine kinase, favouring genomic stability. TTI1 was found to be upregulated in radioresistant tumours [49]. Using a panel of colon cancer cell lines, the authors demonstrated that most radioresistant cells exhibit higher TTI1 expression and that its overexpression in radiosensitive cells reduced their ionising radiation sensitivity. Conversely, knocking down TTI1 in radioresistant cells enhanced their sensitivity to ionising radiation. This was further supported by *in vivo* xenograft experiments, in which TTI1 expression in subcutaneous tumours reduced the efficacy of ionising radiation, thereby limiting tumour growth. Mechanistically, TTI1 appears to activate the ATM signalling pathway, which, in turn, increases DNA repair capacity and decreases apoptosis, thereby contributing to radioresistance [49] (Fig. 1). Interestingly, survivin, a well-characterised inhibitor of caspase-dependent apoptosis, also contributes to DNA damage response by promoting DSBs repair. After ionising radiation (4 Gy), in colon cancer cell lines, Capalbo and colleagues showed that survivin accumulates in the nucleus and interacts with several DNA repair proteins, including Ku70, γ-H2AX, MDC1, and DNA-PKcs, thereby regulating repair activity [50]. In addition, Güllülü et al. demonstrated that the S20 and W67 residues, within the BIR domain of survivin are essential for its interaction with the catalytic domain of DNA-PKcs. This interaction leads to the formation of a heterotetrameric survivin–DNA-PKcs complex with increased kinase activity and enhanced NHEJ repair capacity. These findings suggest that survivin may serve not only as a marker of radioresistance but also as a potential target for radiosensitisation [51] (Fig. 1).

### Reoxygenation

Reoxygenation is the process by which oxygen is redistributed within a tumour, primarily triggering cell death in the more oxygen-rich cells at the tumour periphery. This effect arises because increased oxygen levels enhance ROS generation during exposure to ionising radiation, leading to the formation of DSBs and, consequently, DNA damage. As fractionated radiation therapy progresses, the initially hypoxic and more radioresistant cells present in the tumour core gradually become oxygenated, increasing their radiosensitivity to subsequent ionising radiation doses. Therefore, hypoxia is a key predictor of poor OS and reduced therapy response, as it limits oxygen availability to cancer cells [52] (Fig. 1). In RC, hypoxia plays a central role in mediating resistance to chemoradiotherapy. Huang and colleagues explored this relationship by focusing on hypoxia-inducible factor 1-alpha (HIF-1α), a key regulator of the cellular response to low oxygen levels. Their findings demonstrated that high levels of HIF-1α in RC tissue were significantly associated with aggressive disease, including increased lymphovascular and perineural invasion, higher TRGs, and lower rates of pCR following nCRT. Interestingly, elevated HIF-1α expression was also correlated with hyperglycemia, suggesting a metabolic influence on tumour hypoxia [53] (Fig. 1).

### Redistribution

One of the most important features of cells is their ability to proliferate, a process primarily divided into four phases: G_1_, S, G_2_, and M. The first and longest stage, G_1_, begins immediately after DNA synthesis and can last up to ten hours. During this time, the cell increases in volume and produces precursors for DNA, RNA, and proteins. G_1_ is also a critical decision point, as the cell may continue through the cell cycle, undergo differentiation, enter cell death, or transit into the resting state, G_0_. Notably, the end of G_1_ is considered one of the most radiosensitive stages within the cell cycle. Following G_1_, the cell enters the S phase, which typically lasts 5–10 h. This period is characterised by DNA replication and is considered the most radioresistant stage, as DNA damage repair mechanisms are upregulated. The next stage, G_2_, lasts approximately 1 to 3 h, during which cells gradually become more sensitive to ionising radiation. The M stage is the shortest, lasting only 0.5–1 h, but it is the second most radiosensitive period due to the high level of chromosomal activity [27]. Since cells in different phases of the cell cycle exhibit varying levels of sensitivity to ionising radiation, exposure to a single radiation dose results in unequal effects, leading to the selective death of the more radiosensitive cells. The surviving cells, which are typically in the more radioresistant phases of the cycle, continue to progress, redistributing themselves into the more radiosensitive phases, such as G₂ and M. Consequently, when a subsequent dose of ionising radiation is administered, it becomes more effective at inducing cell death. This principle underlies fractionated radiotherapy, in which multiple smaller doses of ionising radiation are delivered over time rather than a single large dose. By allowing normal tissues to repair sublethal damage between fractions, while increasing tumour cell kill through repeated targeting of radiosensitive phases, this approach enhances the overall therapeutic effectiveness of radiation treatment.

Nevertheless, tumour cells appear to possess mechanisms that subvert cell-cycle-mediated radiosensitivity (Fig. 1). The Maternal Embryonic Leucine Zipper Kinase (MELK) was reported as a potential key player in RC radioresistance. Exposure of the RC cell SNU-503 to a 4 Gy dose of γ-irradiation resulted in a marked upregulation of MELK expression. Conversely, downregulation of *MELK* via siRNA-mediated silencing reduced cell proliferation and led to significant alterations in cell cycle distribution, specifically an accumulation of cells in the G₀/G₁ phase and a decrease in the G₂/M phase. This redistribution suggests a disruption of normal cell cycle progression, which likely contributes to enhanced radiosensitivity observed upon *MELK* inhibition. Furthermore, when siMELK-treated cells were subjected to γ-irradiation 24 h post-transfection, their proliferative capacity was further diminished, providing additional evidence that MELK plays a crucial role in maintaining the cell cycle dynamics that support radioresistance [41]. These findings collectively highlight MELK as a potential therapeutic target for sensitising tumour cells to radiotherapy by interfering with their cell-cycle control mechanisms (Fig. 1).

### Radiosensitivity and Repopulation

The ultimate goal of radiotherapy is to induce tumour cell death or senescence, thereby reducing tumour volume and facilitating surgical resection, a process fundamentally dependent on malignant cells radiosensitivity. In parallel, the healthy surrounding tissues exhibit a capacity for repair and regeneration, as surviving healthy cells repopulate the irradiated area. However, tumour cells frequently acquire adaptive mechanisms that enable them to evade apoptosis and resist radiation-induced cell death, ultimately compromising the therapeutic efficacy of radiotherapy and contributing to radioresistance [28] (Fig. 1).

In RC, variations in the response to ionising radiation have been associated with the balance between pro- and anti-apoptotic protein expression. The X-linked inhibitor of apoptosis (XIAP), a potent inhibitor of caspases-3, −7 and −9, was found to be significantly overexpressed in RC non-responders, whereas DIABLO, a mitochondrial protein that antagonises XIAP, was increased only in good responders [54, 55]. Although activated caspases were consistently upregulated in tumour tissues compared to adjacent healthy mucosa, these changes did not correlate with treatment response. Functional studies have further revealed that silencing XIAP expression significantly enhances radiation-induced apoptosis, suggesting that the XIAP/DIABLO ratio may serve as a predictive biomarker for radiotherapy response in RC. Thus, targeting XIAP emerges as a promising therapeutic strategy to increase radiosensitivity and improve clinical outcomes in XIAP-^high^ tumours (Fig. 1).

Another key regulator is survivin, whose expression was found to be increased in RC-naïve biopsies. The expression of survivin, encoded by the Baculoviral IAP repeat containing 5 (*BIRC5)* gene, was correlated with decreased apoptosis and served as a predictive marker of response to preoperative nCRT. In fact, tumours with elevated survivin levels exhibited a significantly higher risk of local recurrence than those with low survivin expression, underscoring the pivotal role of this molecule in mediating radioresistance and disease progression [56]. Furthermore, downregulation of *BIRC5* mRNA in colon cancer cells resulted in a marked reduction in metabolic activity and clonogenic survival. This effect was accompanied by an accumulation of cells in the G_2_/M phase and by increased γ-H2AX expression, indicative of DNA damage. Additionally, treatment with short-interfering RNA targeting survivin enhanced cell sensitivity to ionising radiation, further supporting the role of this protein in modulating radiotherapy response [56] (Fig. 1).

Another important contributor to RC radioresistance is the Serum and Glucocorticoid-regulated Kinase 1 (*SGK1*) gene. RNA sequencing analysis comparing LARC patient samples with radioresistant or radiosensitive features revealed that SGK1 was among the most upregulated genes in the aldosterone-regulated sodium reabsorption pathway in non-pCR cases compared with pCR. This finding was further validated in an independent cohort of 110 LARC patient samples and in colon cancer cells, where radioresistant models consistently exhibited increased SGK1 expression. These results suggest that SGK1 plays a critical role in mediating cellular adaptation to radiation stress, potentially contributing to the maintenance of survival pathways that underpin tumour radioresistance [57] (Fig. 1).

The regenerating family member 4 (REG4) gene has also emerged as a significant player in RC radioresistance. Its expression was found to correlate with increased survival following γ-irradiation and reduced DNA damage. Importantly, REG4, along with DNA damage repair and apoptosis-related genes, such as the Nei-like DNA glycosylase 2 (*NEIL2)* and BIRC5, was significantly upregulated in tumour samples from LARC non-responders, highlighting its role in mediating resistance to ionising radiation [58].

Transcriptomic profiling studies of radioresistant RC cell lines, including SNU-61R80Gy, SNU-283R80Gy, and SNU-503R80Gy, revealed a marked upregulation of the tumour suppressor gene *N-myc* downstream regulated 1 (NDRG1). Downregulation of NDRG1 in radioresistant SNU-503R80Gy cells led to re-sensitisation to ionising radiation, restoring both active caspase-3 and phosphorylated γ-H2AX to levels comparable to those in SNU-503 naïve radiosensitive cells. Additionally, after 72 h, SNU-503R80Gy parental cells exhibited a significantly higher capacity for DNA damage repair than SNU-503R80Gy shNDRG1 cells, suggesting that NDRG1 plays a crucial role in enhancing the repair mechanisms that characterise the RC radioresistant phenotype [59] (Fig. 1). Complementing these findings, a clinical study involving 86 LARC patients treated with short-course preoperative radiotherapy identified overexpression of the anti-apoptotic human phosphatidylethanolamine-binding protein 4 (hPEBP4) gene in poor responders [60]. Furthermore, elevated hPEBP4 expression was significantly associated with unfavourable tumour regression and increased tumour progression, highlighting that hPEBP4 could serve as a valuable biomarker for predicting radiotherapy response in RC. In fact, the study reported a sensitivity of 55%, specificity of 82%, and accuracy of 76%, reinforcing the potential of hPEBP4 as a predictive marker for radiotherapy outcomes in RC patients [60]. Additionally, Eschrich and colleagues developed a 10-gene Radiosensitivity Index signature (RSI), namely by using Androgen Receptor (AR), Jun proto-oncogene, AP-1 transcription factor subunit (JUN), Signal transducer and activator of transcription 1 (STAT1), Protein kinase C beta (PRKCB), RELA proto-oncogene, NF-kB subunit (RELA), ABL proto-oncogene 1, non-receptor tyrosine kinase (ABL1), Small ubiquitin like modifier 1 (SUMO1), Cyclin dependent kinase 1 (CDK1), Histone deacetylase 1 (HDAC1) and Interferon regulatory factor 1 (IRF1) genes, from gene expression profiles of 48 cancer cell lines and subsequently validated in an independent rectal cancer cohort, receiving neoadjuvant chemoradiotherapy (*n* = 14), showing RSI as a promising indicator for personalized radiotherapy approaches [61].

### Reactivation

The tumour microenvironment (TME) plays a critical role in shaping tumour response to radiotherapy, with various cellular components promoting therapeutic sensitivity or contributing to resistance. While the role of the TME in RC radioresistance remains underexplored overall, several studies have examined how immune cells, fibroblasts, and other stromal components influence RC cells resistance to therapeutic interventions. One such concept is Reactivation, which refers to the immune system activation through the release of tumour-associated antigens following radiotherapy-induced cell death. These antigens are recognised, processed, and presented by antigen-presenting cells, leading to the stimulation of effector T cells and the initiation of an adaptive immune response. In the following sections, under the context of Reactivation, we will review the role of various TME players in RC radiotherapy response (Fig. 1).

#### T lymphocytes

Radiotherapy is known to enhance the infiltration of cytotoxic T cells into the TME, stimulating anti-tumour immunity. However, ionising radiation may have an opposite effect, leading to upregulation of immune checkpoint molecules such as PD-L1, CTLA-4, and LAG3, thereby promoting immunosuppression and tumour growth [62].

Tumour infiltrating lymphocytes (TILs) are determinant to an efficient therapeutic response. In biopsies prior to nCRT, CD4^+^ and CD8^+^ TIL density correlated significantly with tumour response. Higher TILs densities were associated with better tumour regression, with CD8^+^ TIL density an independent prognostic factor for complete response [63]. Similarly, Yang et al. reported that increased numbers of cytotoxic CD8^+^ T lymphocytes correlated with better pathological responses to nCRT in LARC [64]. In addition, biopsy analysis of LARC patients who received nCRT showed that CD4^+^ and FOXP3^+^ TILs density was significantly correlated with TRG and with tumour shrinkage rate [65]. Single-cell RNA sequencing of LARC samples, before and after nCRT, revealed that non-responders exhibit a predominantly immunosuppressive TME, characterised by altered macrophage-T cell interactions and ECM-driven fibroblast remodelling, suggesting that cellular reprogramming post-treatment contributes to resistance [66].

A very elegant study, led by OʼBrien and colleagues, explored the role of complement C3 and C5 proteins, which play a central role in immune system activation towards radioresistance [67]. Interestingly, the authors found that levels of these complement proteins were lower in the more radiosensitive HCT116 colon cancer cell line than in the more radioresistant SW837, SW1463, and HRA-19 RC cell lines. This was also observed for the cleaved, smaller fragments C3a and C5a. Additionally, upon exposure to 1.8 Gy, HCT116 (the more radiosensitive cell line) and C3-overexpressing cells showed a significant increase in radiation resistance. Concomitantly, exposure of the transiently C3-silenced HRA-19 RC cell line (the more radioresistant) to 1.8 Gy of ionising radiation resulted in a significant reduction in cell survival and a significant increase in DNA damage [68]. At 24 h post-transfection, C3 silencing was associated with a significant decrease in the percentage of cells in S phase and an increase in the percentage of cells in G_2_M phase, suggesting cell cycle arrest.

Additionally, the levels of C3 and C5 proteins were assessed in naive RC (*n* = 18) and in healthy rectal tissue biopsies (*n* = 20) from patients undergoing investigative colonoscopy. RC samples showed significant upregulation of C3 and C5 mRNA compared with heathy tissue (Fig. 1). Moreover, expression of the Complement Factor B (*CFB*), a marker of the alternative pathway of activation, was significantly upregulated in tumour tissue. Additionally, serum levels of complement components were elevated and associated with poor responses to nCRT and poor survival in RC patients [68] (Fig. 1).

By analysing patient serum, Liu et al. found that senescence-associated secretory phenotype (SASP) factors and post-nCRT immune cell infiltration could predict nCRT response. This study was performed in patients who underwent a standardised nCRT regimen with a total ionising radiation dose of 50.4 Gy in 28 fractions, combined with concurrent Xeloda/capecitabine-based chemotherapy (*n* = 20). The results indicated that IL-1α, IL-6, IL-8, C-reactive protein (CRP), CCL5, CCL2 and CXCL1 levels were higher after nCRT, suggesting an inflammatory response mediated by treatment. Moreover, immunohistochemistry analysis unveils a significant upregulation of CD8^+^ cytotoxic T cells and of macrophages expressing the mannose receptor, CD206. However, no correlation between cytokine/chemokine up-regulation and poor response to nCRT was found. Only after nCRT, responders show a significant increase in infiltration of CD8^+^ cytotoxic T cells in the TME, whereas no change was observed in non-responders [69]. In a very recent study, Wang and colleagues used 15 LARC patient biopsies and post-treatment resections following nCRT. They observed a distinct population of early-stage exhausted T cells (early-Tex) NR1D2^+^, and more abundant Tertiary lymphoid Structures (TLS) increased in responders, concomitantly with reduced immunosuppressive signals, findings that are crucial for patients stratification strategies [70] (Fig. 1).

#### Macrophages

TAMs have also emerged as central players of the immune landscape within the TME, often contributing to immune suppression and radioresistance [71, 72]. Indeed, Kamran et al. and Seo et al. observed that M2-like macrophages were significantly higher in post-nCRT samples compared with pre-treatment samples, and Yang and colleagues demonstrated that increased TAM infiltration was associated with poor therapeutic outcomes, emphasising their role in treatment failure [64, 73, 74]. Moreover, carcinoembryonic antigen (CEA), a biomarker commonly increased in RC, was shown to promote TAM-mediated radioresistance by directing macrophage polarisation toward a pro-tumour M2-like anti-inflammatory phenotype. The M2-like macrophage phenotype, characterised by its immunosuppressive and anti-inflammatory functions, secretes immunomodulatory mediators, such as prostaglandin E₂ (PGE₂), which fosters a protective TME and confers resistance to radiation-induced cell death [75]. Furthermore, mast cell infiltration, which facilitates TAM recruitment, has been correlated with poor prognosis and reduced responsiveness to nCRT in RC, highlighting the complex interplay among immune cell populations in shaping tumour behaviour and in modulating treatment efficacy [76] (Fig. 1).

#### Fibroblasts

In addition to the immune compartment, cancer-associated fibroblasts (CAFs) are crucial for communicating and shaping the extracellular matrix and the overall TME structure, and cancer cells, thereby driving metastatic progression [77]. In 2011, Saigusa and colleagues were the first to correlate the expression of fibroblast activation protein alpha (FAP-α) with the secretion of CXCL12 in residual RC stroma after nCRT and distant recurrence, and to show that this association was associated with poor prognosis [78] (Fig. 1). Later, Harpain et al*.* observed increased expression of Fibroblast growth factor-8 (FGF8) in RC biopsies from non-responders compared with nCRT responders [79] (Fig. 1). Additionally, Tommelein and colleagues reported that CAFs may play an important role in affecting the sensitivity of the neighbouring cancer cells. By irradiating CAFs with clinically relevant fractioned regimens (1.8 Gy for 1 day, 1.8 Gy for 5 consecutive days and 1.8 Gy for 10 consecutive days), the authors were able to demonstrate that radiotherapy-treated CAFs secrete paracrine-acting factors that change the metabolic activity of colon cancer cells (COLO320DM) by increasing glucose and glutamine consumption, promoting their survival. Meanwhile, Insulin-like Growth Factor 1 (IGF1) secretion by irradiated CAFs activated the IGF1R-Akt-mTOR signalling pathway, which, when neutralised *in vivo,* in colon orthotopic xenograft models, significantly reduced metastatic burden [80] (Fig. 1).

Inflammatory CAFs (iCAFs) have been shown to play a significant role in driving radioresistance. Their ability to secrete inflammatory cytokines, as IL-1, triggers oxidative DNA damage in stromal cells, inducing their senescence post-irradiation [81]. This senescence fosters a pro-tumourigenic environment that enhances resistance to nCRT. Similarly, by secreting exosomes that mediate intercellular signalling, iCAFs enhance cancer stem cell-like traits in RC cells, supporting tumour resistance to ionising radiation [82] (Fig. 1). Another report showed that higher CXCL12 expression in pre-nCRT biopsies of LARC patients was associated with radioresistance, tumour recurrence, and infiltration of pro-tumour populations, namely regulatory CAFs and CD4^+^ T cells. Increased CXCL12-CXCR4 signalling after radiotherapy was reported to promote tumour radioresistance, suggesting that a CXCL12-CXCR4 inhibitor is a promising strategy to overcome radioresistance [83–85] (Fig. 1).

### Reprogramming

Metabolic alterations have emerged as a hot topic in cancer research, particularly in the context of therapy resistance, including to radiotherapy. Cancer cells are known to reprogram their metabolic pathways to adapt to the stressful conditions induced by ionising radiation, becoming able to survive and evade radiotherapy-induced cell death. Key metabolic pathways commonly affected include those involved in nucleotide, lipid, iron, glutamine, and glucose metabolism. These alterations confer a significant advantage to cancer cells, enabling them to thrive in an otherwise catastrophic microenvironment, where normal cellular processes are disrupted by radiation-induced damage [86–88]. Therefore, based on increasing evidence of metabolic rewiring in cancer cells that resists radiotherapy-induced cell death, we propose a 7th R in radiobiology: *Reprogramming* (Fig. 1).

The inclusion of the 7th R is supported by several studies, including a comparison between the more radioresistant RC cell line (SW837) and the more radiosensitive colon cancer cell line (HCT116) under standard, hypoxic, and 5-FU-normoxia conditions. This study revealed significant alterations in canonical pathways that are associated with radiotherapy response, including cell cycle regulation, cellular metabolism, oxidative stress, and DNA damage repair. Oxidative phosphorylation was the most significantly altered metabolic mechanism in the transcriptome of radioresistant SW837 cells compared with that of radiosensitive HCT116 cells, highlighting a potential metabolic phenotype associated with radioresistance. Additionally, alterations in 16 metabolites involved in the phosphatidylcholine metabolic pathway in the pre-treatment serum of RC patients were associated with poor pathological response to nCRT and with worse recurrence-free and OS, supporting their potential as circulating predictive biomarkers of treatment response in RC [89]. Furthermore, metformin, a clinically approved inhibitor of oxidative phosphorylation for the treatment of type 2 diabetes, was administered to SW837 and HCT116 cells, resulting in significant alterations in energy metabolism and mitochondrial function. This included reduced oxidative phosphorylation, increased ROS production, and changes in mitochondrial membrane potential (Fig. 1). In SW837, a significant increase in the gluconeogenesis pathway and mitochondrial mass was observed. The radiosensitising effects of metformin at concentrations of 1, 2.5, and 10 mM were evaluated in both cell lines exposed to the clinically relevant dose of 1.8 Gy of X-ray ionising radiation. The clonogenic assay, considered the gold standard for assessing cell survival, revealed that metformin significantly enhanced radiosensitivity in both cell lines by altering cell cycle phase distribution and inducing apoptosis. Furthermore, metformin treatment on naïve *ex vivo* RC patients biopsies showed a marked reduction in metabolic activity, evidenced by a 47% decrease in oxygen consumption and a 36% reduction in extracellular acidification rates [90]. Of note, since these cell lines are from distinct tumour types and have different genetic backgrounds, it is possible that these metabolic changes are not exclusively related to ionising radiation effects.

Another transcriptomic analysis using the RC dataset GSE35452 identified Intelectin 1 (ITLN1*)*, a gene potentially involved in glucose uptake, associated with decreased response to nCRT. Immunohistochemical staining of tumour samples from a cohort of 343 patients revealed that high ITLN1 expression was significantly correlated with adverse pathological features, including advanced post-treatment tumour stage (T3–4), nodal involvement (N1–2), as well as vascular and perineural invasion. Moreover, elevated ITLN1 levels were significantly associated with poor TRG following nCRT, supporting its potential role as a prognostic biomarker [91] (Fig. 1). More recently, Ferrandon S.et al. showed that CoA synthase (COASY), a bifunctional enzyme that catalyses the last two steps of CoA synthesis and that is crucial for tricarboxylic acid cycle (TCA) activity, was significantly higher in stage II and III tumours from 33 RC patients who underwent nCRT than in healthy tissue [92]. COASY overexpression was associated with poor radiotherapy response, establishing this protein as a reliable predictive marker to distinguish radiotherapy responders from non-responders. The molecular mechanism underlying COASY-mediated radioresistance involves its interaction with the PI3K regulatory subunit p85α, which subsequently activates the PI3K/Akt signalling pathway. This pathway plays a crucial role in cell survival mechanisms in RC, particularly in response to radiotherapy. Downregulation of COASY results in reduced PI3K/Akt pathway activation, leading to impaired DNA DSBs repair and subsequent increase in radiosensitivity [92–95]. Notably, COASY was found to modulate DNA repair efficiency in both homologous recombination and NHEJ-proficient cells. COASY downregulation reduced the double-strand break repair nuclease (*MRE11*) levels in RKO cells (homologous recombination-proficient) and decreased the levels of DNA-PKcs and Ku70 in HRT-18 cells (homologous recombination-deficient). Additionally, in a CRC xenograft mouse model, COASY inhibition sensitised the tumour to radiotherapy, reducing tumour growth kinetics. This supports its role in RC radioresistance via PI3K/Akt pathway activation, DNA-PKcs, Ku70, and MRE11 recruitment to DSBs sites, powering DNA damage repair [92] (Fig. 1).

Within the concept of metabolic reprogramming, alterations in lipid metabolism have also recently gained significant attention. The previously described PI3K, the phosphoinositide 3-kinase, is a major modulator of lipid metabolic pathways and a facilitator of proto-oncogenic bioactive lipid-mediated signalling [96]. A hyperactive PI3K pathway enhances lipid biosynthesis by upregulating SREBP1 and its target enzymes FASN, ACC, and SCD1. This increases *de novo* fatty acid and cholesterol synthesis, remodulates energy storage, and reshapes lipid metabolic pathways, further modulating ROS responses and DNA repair signalling, ultimately contributing to radioresistance [97] (Fig. 1). The analysis of 11 paraffin-embedded rectal tumours, five radioresistant vs six radiosensitive, revealed that Phosphatidylinositol transfer protein cytoplasmic 1 (PITPNC1) transcripts and protein levels were significantly higher in patients who relapsed after radiotherapy. Modulating PITPNC1 expression in colon cancer cells, submitted to radiotherapy was associated with higher PITPNC1 expression and lower ROS production. Conversely, inhibition of PITPNC1 decreased cell proliferation, while increasing apoptosis and ROS levels. Notably, this effect was reversed upon treatment with N-acetylcysteine (NAC), a potent ROS scavenger [82], highlighting the role of PITPNC1 in regulating oxidative stress and its potential as a target to enhance radiosensitivity (Fig. 1). In another study, the lipid transporter ATP-binding cassette subfamily C member 4 (ABCC4) was identified as a potential mediator of radioresistance in RC and showed prognostic relevance. ABCC4 expression in biopsy samples was positively correlated with a poor short-term pathological response to nCRT in patients with LARC [98]. Moreover, high ABCC4 expression, combined with *TP53* mutations, was significantly associated with worse 3-year OS and DFS in pre-treated samples. Notably, in a xenograft model, ABCC4 inhibition led to a significant increase in intracellular cAMP levels following 4 Gy ionising radiation, suggesting interference with multiple signalling pathways. This was further accompanied by the accumulation of cells in the G_2_ phase, the most radiosensitive cell cycle stage, supporting a mechanistic link between ABCC4 activity, cell cycle regulation, and radioresistance [98] (Fig. 1).

## Transcriptional plasticity: cancer stem cells

Radiotherapy can also be a strong transcriptional stressor. In resistant tumour cells, radiotherapy can promote rapid changes in gene expression, enabling phenotypic adaptation [99]. Cancer stem cells (CSCs), often considered the “seeds” of cancer development, have also been associated with radioresistance, greatly influencing cells transcriptional capabilities [100]. Although these cells represent a small fraction of the tumour burden, they exhibit enhanced DNA repair mechanisms and antioxidant defences, and can remain quiescent while maintaining rapid cell renewal [101]. The B lymphoma Mo-MLV insertion region 1 homolog (BMI1) is a polycomb group protein frequently overexpressed in several cancer types that plays a key role in the epigenetic regulation of CSC self-renewal and stemness [102, 103]. Increased BMI1 expression positively correlated with advanced tumour stage and tumour progression, as demonstrated in a study comprising 172 RC patients treated with nCRT [104]. Silencing *BMI1* genes in colon cancer cells enhanced sensitivity to radiotherapy and 5-Fluorouracil-based chemotherapy, while impairing cellsʼ ability to form spheres, a key indicator of stem-like properties and tumour-initiating capacity. Accordingly, BMI1 silencing reduced the mRNA levels of several stem cell markers, such as the SRY-box transcription factor 2 (SOX2*)*, the POU class 5 homeobox 1 (OCT4*)*, the Nanog homeobox (NANOG*)* and the KLF transcription factor 4 (KLF4*)*, which are closely related to RC recurrence and poor prognosis [41]. Among the referred molecules, OCT4 overexpression is known to be associated with increased resistance to ionising radiation in a dose-dependent manner [105]. In addition, increased ectopic OCT4 expression in SW480 colon cancer cells enhanced tolerance to radiotherapy, as they recovered more quickly from the G_2_ cell cycle phase 48 h after ionising radiation. Overexpression of OCT4 led to upregulation of Zinc finger E-box binding homeobox 1 (ZEB1), N-cadherin, and vimentin, and downregulation of E-cadherin [105]. These epithelial-mesenchymal transition (EMT) markers are closely linked to therapy resistance, including radioresistance, as they confer stem-like properties to tumour cells, enhancing their plasticity and ability to evade treatment [106, 107]. Additionally, OCT4 overexpression in SW480 colon cancer cells led to a faster reduction in γ-H2AX levels after 3 Gy of ionising radiation [89], whereas ZEB1 inhibition attenuated the radioresistance induced by OCT4. This was accompanied by a downregulation of the Checkpoint kinase 1 (CHK1), which affected RC cells ability to repair radiation-induced DNA damage [105].

### Non-coding RNAs: key regulators of radioresistance in RC

Regulatory proteins play a major role in cancer therapy resistance; however, only 2% of the human genome is expressed as proteins. Approximately 98% of the genetic material is non-coding, with the majority still transcribed to RNA [108]. Non-coding RNAs (ncRNAs) were originally thought to be an artefact of transcription and consequently non-functional. However, this notion has shifted, and an extensive body of work has emerged exploring the biological functions of ncRNAs in health and disease. These non-coding fragments have emerged as important epigenetic regulators in cancer, with many becoming aberrantly expressed as the disease progresses [109]. Some of these ncRNAs can reach the bloodstream and potentially serve as biomarkers for cancer diagnosis and prognosis [94, 95], while others can modulate radiotherapy.

The ncRNA class includes small nuclear RNAs (snRNAs), which participate in messenger RNA (mRNA) splicing processes, transfer RNAs (tRNAs), which organise mRNA translation into protein synthesis, and ribosomal RNA (rRNA), the largest portion of RNA responsible for translation [110]. However, in the oncology field, most of the recent work on ncRNAs has focused on regulatory RNAs such as: (i) micro RNAs (miRNAs), (ii) small (≈20 nucleotide long) fragments that bind to mRNA and suppress their expression, and (iii) long non-coding RNAs (lncRNAs), which exhibit a plethora of modes of action, as sponging miRs or stabilizing mRNAs, to regulate gene expression [111].

**Fig. 2:**
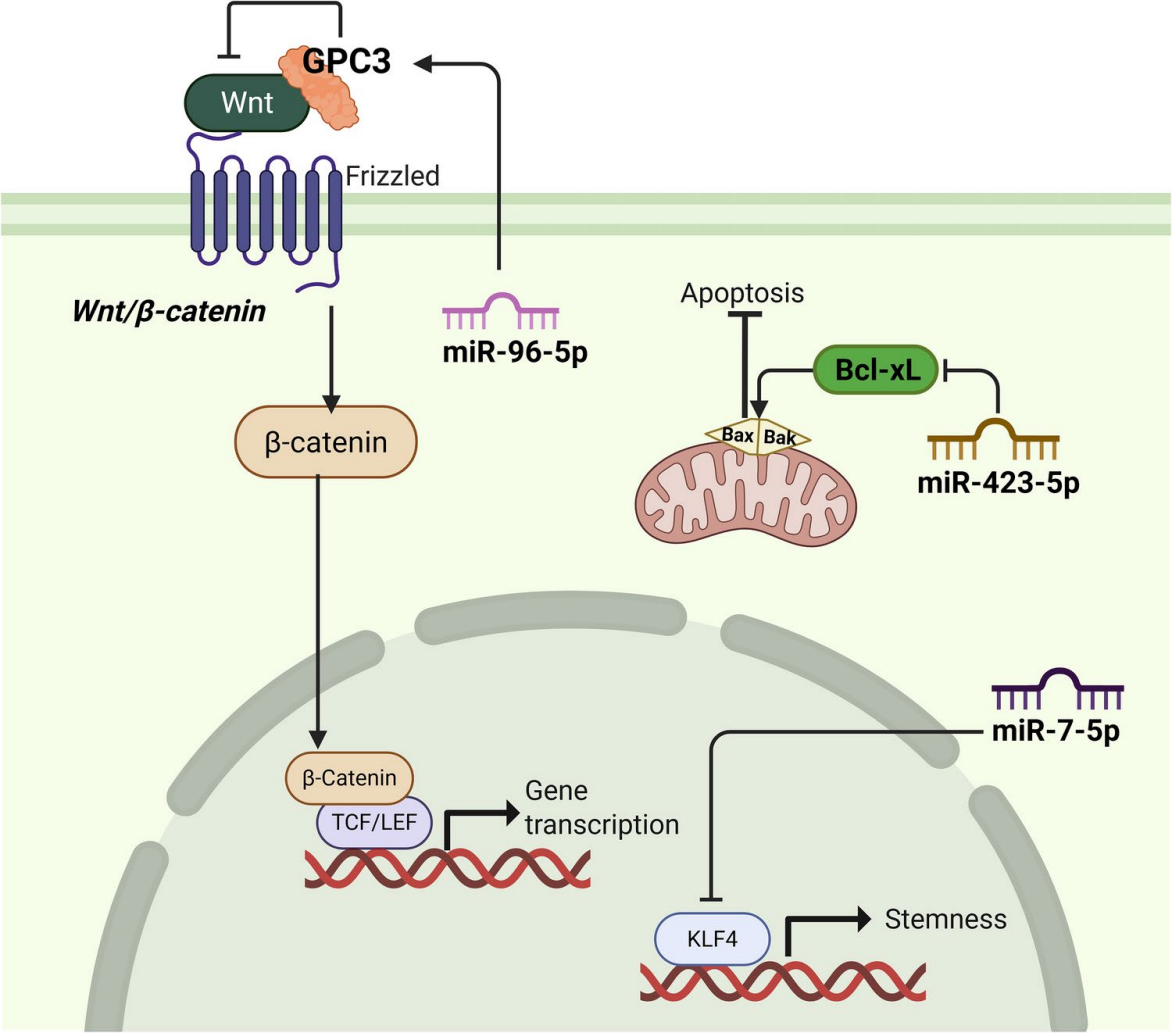
Overview of the miRNA ecosystem in RC radioresistance. Cancer cells employ several mechanisms to survive the stress imposed by ionising radiation, among which is the expression of miRNAs that target essential survival pathways. The previously identified RC-associated miR-96-5p is known to interact with GPC3, inhibiting the Wnt/β-catenin signalling pathway and, subsequently, affecting the transcription of stemness-associated genes. This is also the case of miR-7-5p, which inhibits KLF4, thereby unlocking the transcription of stemness-related genes. miR-423-5p, when decreased in cancer cells, promotes the Bcl-xL survival pathway and decreases apoptotic levels. Altogether, these multiple interactions favour cancer cell survival and contribute to RC radioresistance

MiRNAs regulate gene expression through binding to complementary 3′ untranslated regions (UTR) of mRNAs, blocking their translation and targeting them for degradation [112]. As epigenetic regulators in cancer, they gain special importance when they assist tumour progression and therapy resistance. In RC, a screening of radioresistant and radiosensitive tumours in a PDX mouse model revealed four miRNAs overexpressed in the radioresistant subset: miR-552-5p, miR-182-5p, miR-183-3p and miR-96-5p. The latter was also identified in five different RC cell lines, relating to their capacity to resist radiotherapy. Additionally, lentiviral transfection inhibiting miR-96-5p in HR-8348 cells resulted in increased GPC3 expression, inhibited the Wnt/β-catenin signalling pathway, and decreased the radioresistance phenotype [113] (Fig. 2). A study by Shang et al. revealed that miR-423-5p can modulate apoptosis and enhance radioresistance. Using two parental colon cancer cell lines (HCT116 and RKO) and their radioresistant derivatives (HCT116-R and RKO-R), the authors compared miRNA signatures and found that miR-423-5p was downregulated in the radioresistant cells, which displayed decreased apoptosis following 4 Gy of ionising radiation (Fig. 2). This miRNA was subsequently identified as a Bcl-xL suppressor, and thus its downregulation in radioresistant cells revealed pro-apoptotic functions of miR-423-5p [114] (Fig. 2). In this study, the authors identified another miRNA, miR-7-5p, as significantly decreased in radioresistant cells compared to their parent cells [115]. The presence of miR-7-5p is associated with higher survival rates and lower proliferation following radiotherapy, and its expression is related to a decreased stemness phenotype. miR-7-5p was shown to suppress stem cell-like behaviour, promoting cell resistance to therapy through inhibition of *KLF4* expression, a stemness-related gene.

Furthermore, miR-7-5p was also found to be downregulated in non-responder RC patient biopsies and, by establishing a PDX RC mouse model derived from a LARC patient resistant to radiotherapy, the authors demonstrated the ability to radiosensitise tumours via delivery of miR-7-5p-loaded nanoparticles [116] (Fig. 2). Additionally, the same authors reported that miRNA-130a was downregulated in the more radioresistant RC cells (SW837 and SNU1411) compared with the more radiosensitive ones (SNU-70 and SW1463). Overexpression of miRNA-130a in highly radioresistant cells sensitised them to ionising radiation by targeting SOX4 and by suppressing ATM-dependent DNA damage repair signalling, as corroborated in an RC xenograft model [117].

Apart from miRNAs, lncRNAs have also been shown to regulate the response to RC radiotherapy. Accordingly, Kutilin et al. showed that interactions between miRNAs and lncRNAs regulate the expression of genes encoding DNA repair and apoptosis-related proteins, including γ-H2AX, RAD50, RBBP8, BCL2, BRCA-2 and Caspase 9. Good responders exhibited overexpression of Caspase 9 and downregulation of γ-H2AX and RBBP8, which are associated with impaired DNA repair and enhanced apoptosis. Conversely, partial or non-responders showed upregulation of BCL2, γ-H2AX, RAD50, RBBP8, and BRCA2, promoting efficient DNA damage repair and resistance. Notably, low expression of lncRNAs, including XIST, NEAT1, HELLPAR, and AC008124.1, in responders reduced miRNA sequestration, increasing the pool of active miRNAs capable of downregulating pro-survival and repair genes. These findings underscore the regulatory potential of ncRNAs as biomarkers and modulators of therapeutic response in RC [118].

## Advances in experimental models for studying RC radioresistance

### Cell lines as pivotal models in RC research

**Fig. 3:**
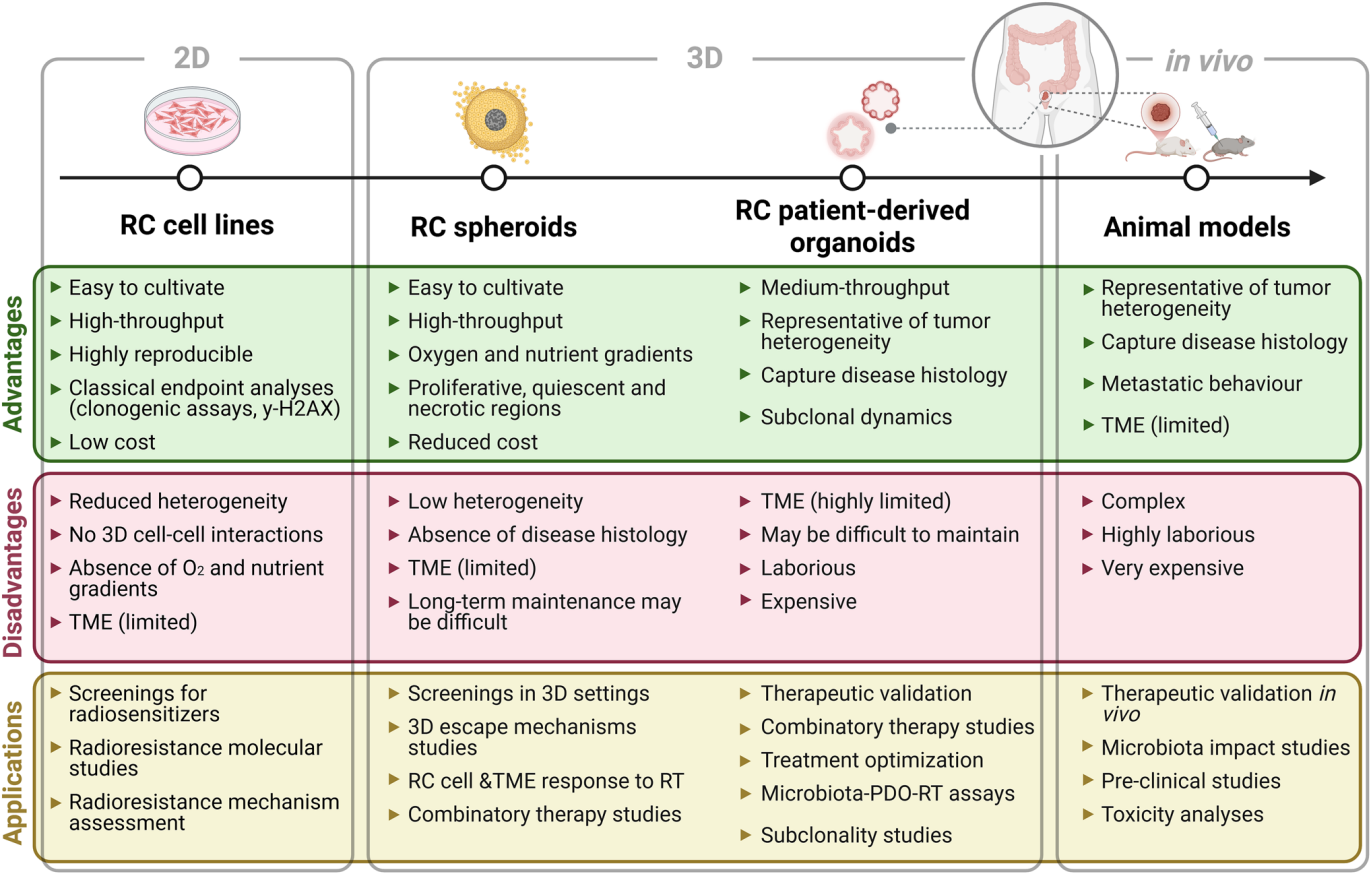
Models of RC to advance radioresistance research. There are multiple models for studying RC radioresistance, yet they all have different advantages and disadvantages. Cell lines are easy to cultivate and can be used in high-throughput assays to perform multiple screenings. Spheroids exploit a 3D structure to mimic tumour morphology and physiological features, including oxygen and nutrient gradients. These 3D models are easy to cultivate and enable high-throughput analysis. Organoids have recently been widely used because they preserve tumour heterogeneity, disease histology, and subclonal dynamics. However, they only support medium-throughput assays and are more labour-intensive. To advance complexity, animal models are recommended. These models exhibit most tumour features and often mimic the human disease. However, the immune compartment remains a hurdle, and treatment with ionising radiation requires specialised equipment, such as SARRP (Small Animal Radiation Research Platform) units

Patient-derived cell lines played a crucial role in advancing cancer research. Among these, monolayer cultures stand out as a simple and cost-effective approach to address the significant challenge of radioresistance in RC treatment. By leveraging immortalised cell lines derived from primary or metastatic tumours, morphological and molecular characteristics are preserved, making them particularly useful tools for investigating radioresistance mechanisms (Fig. 3). In RC, the SW837 and SW1463 cell lines are widely used, well-established models developed by Dr A. Leibovitz in the 70 s [119]. Based on the American type culture collection, the SW837 cell line was derived from a 53-year-old Caucasian male patient diagnosed with Grade IV rectum adenocarcinoma. These cells are categorised as part of the inflammatory subtype, exhibit a microsatellite-stable (MSS) phenotype, are K-RAS (G12C) and p53 (C248T) mutated, and express PTEN. The SW1463 cell line was established from a 66-year-old Caucasian female patient with rectal adenocarcinoma, classified as Dukesʼ type C, derived from a grade II mucous-producing tumour with grade III solid tumour characteristics. It is classified as the transit-amplifying subtype, a distinct molecular subtype with unique biological behaviour and therapeutic implications, and it holds MSS status, G12C K-RAS mutations, and PTEN expression. Notably, both RC cell lines exhibit an epithelial-like morphology, grow slowly, form adherent populations in tightly packed islets, and are known to produce the CEA. Interestingly, SW1463 cells are mucous-producing and possess brush borders, further highlighting their differentiation characteristics [120, 121]. Korean researchers developed another set of RC cell lines, which were collected from RC specimens obtained during surgeries conducted at Seoul National University (SNU) Hospital from 1988 to 2001 [122]. In their study, five RC cell lines were established and characterised for their phenotypes: SNU-70, SNU-254, SNU-796, SNU-977, and SNU-1411. The SNU-70 and SNU-254 cell lines, derived from well-differentiated tubular adenocarcinomas, exhibited an ulcerofungating and ulceroinfiltrative phenotype, respectively. Both grew as adherent populations, forming dense monolayers of tightly packed epithelial cells. SNU-796 and SNU-977, originating from moderately differentiated adenocarcinomas, displayed ulcerofungating and polypoid phenotypes, respectively, and proliferated as monolayers consisting of epithelial cell islands. The SNU-1411 cell line, also derived from a moderately differentiated adenocarcinoma with an ulceroinfiltrative phenotype, developed as adherent populations and eventually formed dense monolayers. All these cell lines exhibit an MSS phenotype and p53 mutations, but only SNU-1411 harbours a K-RAS mutation (G12D) [122]. To effectively use these cell lines as tools for investigating mechanisms of radioresistance, it is essential first to understand their response to radiotherapy. The cell lines SW837, SW1463, SNU-70, and SNU-1411 exhibited distinct levels of radiosensitivity. In fact, when using the standard colony formation assay, they demonstrated progressively lower sensitivity to ionising radiation at doses ranging from 2 to 9 Gy. Among them, SNU-70 was the most sensitive, followed by SW1463, SW837, and SNU-1411 [117, 123, 124]. To further dissect radioresistance mechanisms, derivative resistant cells were generated through stepwise ionising radiation. For SNU-70, a two-step process involving single doses of 6 Gy was applied, allowing researchers to isolate a surviving population and establish the radioresistant derivative SNU70RR [117]. Similarly, SW1463 cells were subjected to multiple exposures to 2 Gy, totalling 68 Gy, resulting in the development of a SW1463-resistant derivative, which is an invaluable tool for radioresistance biology studies [123]. These cell line RC models facilitated significant progress in understanding how cancer cells adapt to ionising radiation, providing insights into mechanisms such as DNA repair, cell cycle regulation, and survival signalling pathways. However, while monolayer cultures have been instrumental, they are limited in their ability to mimic the complex TME and cellular heterogeneity observed *in vivo*. To bridge this gap, 3D culture systems, including organoids and spheroids, are increasingly being adopted as advanced models for studying RC radioresistance.

### Emerging three-dimensional models in RC research

Three-dimensional cell culture models, as spheroids and organoids, have revolutionised cancer research by providing physiologically relevant systems that replicate key features of tumours. These systems better replicate tumour architecture, cell–cell interactions, the microenvironment, and hypoxic gradients, enabling more accurate studies of tumour biology, therapeutic response, and resistance mechanisms [124–126].

#### Spheroids as powerful tools for RC radioresistance understanding

Spheroids have emerged as a critical model in cancer research, particularly in the context of RC, where they serve as advanced alternatives to traditional 2D cell cultures that often fail to replicate the complexity of tumours [124–126]. These 3D structures can be generated by multiple methods, including ultra-low attachment plates, liquid overlay, or hanging drop techniques (Fig. 3). Despite the diversity of spheroid formation methods, the culture medium and cell seeding density are crucial factors influencing their development. Compact spheroids larger than 300 µm will likely generate metabolic and oxygen gradients that resemble those found in tumours, including the formation of a necrotic core. This core is critical for understanding the heterogeneous nature of tumours, as it influences cell proliferation, quiescence, and apoptosis in the spheroidʼs inner regions [125, 127]. Therefore, optimising both culture medium and seeding density is pivotal for creating spheroids that replicate the structural complexity and physiological properties of tumours. To standardise experimental conditions and enhance the reproducibility of spheroid research, the MISpheroID knowledgebase was established. This resource provides guidelines and essential parameters for conducting spheroid experiments, addressing the variability in spheroid setup and characterisation across different research labs. By offering a structured framework, MISpheroID ensures that the methodological differences in spheroid research do not compromise the transparency and reproducibility of results [128, 129]. Building upon these guidelines, Estêvão and colleagues developed and characterised two RC spheroid models using SW837 and SW1463 cell lines to study the molecular mechanisms underlying radiotherapy responses, also demonstrating that optimising cell seeding density and culture conditions significantly affected spheroid morphology, viability, and the formation of a necrotic core [124]. Additionally, the study demonstrated that SW837 spheroids exhibited greater radioresistance than SW1463 spheroids and highlighted distinct metabolic and transcriptomic profiles in response to a clinically relevant short-course radiotherapy scheme [124]. In addition to its characterisation as a valuable model for radiobiology research, the SW837 spheroid model was integrated into the Spheroid Light Microscopy Image Atlas (SLiMIA), which houses over 8000 images accompanied by extensive metadata. This resource is designed to serve as a comprehensive training set for morphological analyses, facilitating the study of spheroid morphology and growth dynamics under different experimental conditions [130, 131]. Altogether, the SW837 spheroid model holds promise as a cornerstone for future studies focused on RC radioresistance and opens new possibilities for RC pre-clinical research.

#### Biomimetic RC patient-derived organoids (PDOs)

PDOs have emerged as powerful tools for investigating tumour heterogeneity and therapeutic resistance (Fig. 3). In RC, disease-specific pre-clinical organoid models have only recently been developed.

The study led by Ganesh et al. established a biorepository of 65 PDOs from RC patients with primary, metastatic or recurrent disease. These PDOs preserved the molecular and drug sensitivity characteristics of their original tumours, correlating with clinical responses. Moreover, when engrafted in mice, these models recapitulated tumour invasion and metastasis, providing a robust platform for studying RC [132]. Another group generated PDOs from patient-derived xenografts (PDX) obtained from stage II and III patient biopsies, collected prior to therapy [133]. Again, both PDX and PDOs were shown to replicate the mutational profile, disease histology, and chemotherapy response [133]. The potential of PDOs to predict individual responses to nCRT therapy was also assessed using organoids derived from biopsies of patients with LARC. The organoids were subjected to chemoradiation treatments *in vitro*, and their responses were compared to the clinical outcomes of the corresponding patients. The findings indicated that PDOs closely mirrored patients clinical responses, with an accuracy of 84.43%, sensitivity of 78.01%, and specificity of 91.97% [134].

Organoids derived from irradiated RC tumours of poor responders to nCRT were also subjected to chemotherapy sensitivity testing, and the results demonstrated that these 3D models could effectively identify patients likely to benefit from further postoperative chemotherapy. This approach enables the design of tailored adjuvant treatment strategies for patients with residual disease [135]. Since the focus of this review is radioresistance in RC, it is essential to discuss the work performed by M. Park and colleagues [136]. The authors developed PDOs from treatment-naive RC patients to assess their potential in predicting individual responses to radiotherapy. The organoids were subjected to *in vitro* ionising radiation, and the findings indicated a positive correlation between organoid ionising radiation responses and patients actual treatment outcomes.

In line with this, Wanigasooriya et al. investigated the mechanisms driving radioresistance using PDOs from both pre-treatment biopsies and post-treatment surgical specimens. Comparing transcriptomic profiles, an upregulation of the PI3K/AKT/mTOR pathway was identified in radioresistant PDOs. Importantly, subsequent treatment with dual PI3K/mTOR inhibitors enhanced radiosensitivity, suggesting this pathway as a potential therapeutic target to overcome radioresistance in RC [95].

More recently, Xiao and colleagues developed patient-derived RC organoids with different radiosensitivities as a screening platform for 1596 drug-radiation combinations. With the screening, the authors have found that inhibitors of rat sarcoma virus/mitogen-activated protein kinase (RAS-MAPK) enhance ionising radiation response by selectively downregulating RAD51, a component of the homologous recombination DNA repair pathway [137]. In conclusion, PDOs provide a robust platform for investigating the molecular mechanisms underlying treatment responses, particularly in addressing the challenges posed by radioresistance. Their ability to predict individual responses to chemoradiation demonstrates their potential to assist in clinical decision-making, especially for radioresistant patients. Moreover, these 3D models enabled researchers to dissect the molecular mechanisms underlying RC radioresistance, providing insights into key pathways underlying therapy failure. This opens new avenues for testing targeted therapies and stratifying patients based on their tumour sensitivity to treatment.

### Animal models: a cornerstone for RC experimental research

Animal models play a crucial role in RC research, providing an *in vivo* platform to study disease mechanisms and therapeutic responses, particularly when clinical questions are difficult to model in the laboratory. Among the most widely explored models are transplantable tumour cells, xenografts of human carcinomas, and chemically induced tumours in mice. To our knowledge, the first murine model of RC was described in the literature in 1992. It involved the intrarectal injection of tumour cells into immunodeficient mice, enabling localised tumour growth. Researchers injected murine-derived CT26 and MCA38 and human LS174T colon cancer cell lines into the submucosal layer of the rectal wall in nude mice. The model successfully produced tumours confined to the rectum, displaying histological features closely resembling human RC, including local tissue invasion and, in some cases, early metastatic behaviour [138]. Later in 2002, Chen and colleagues reproduced the orthotopic RC mouse model, using CT26 cells in immunocompetent BALB/c mice. In this study, all animals died around day 44 after cell inoculation, suffering from severe cachexia and rectal lumen compression, although no liver or lung metastases were observed [139]. A few years later, another model was developed using CT26 cells in immunodeficient nude mice. In this work, animals successfully developed primary rectal tumours and spontaneous metastases in the tail lymph nodes and lungs [140].

Nevertheless, these results were obtained in the absence of a fully functional immune system, which might have facilitated the metastatic spread of CT26 cells in immunodeficient nude mice, in contrast to the immune-competent BALB/c model. Subsequent models combined the use of chemical agents (Dextran sulfate sodium or 2,4,6-Trinitrobenzenesulfonic acid) to induce colitis, with the implantation of CT26 colon cancer cells in the rectal mucosa of BALB/c animals. This approach resulted in a 90% incidence of tumours at the intraluminal mucosal region, effectively mimicking human RC macroscopically [141]. Furthermore, the authors attempted a similar approach in Severe Combined Immunodeficiency (SCID) mice, combining colitis induction with the intrarectal instillation of human LS174T cells embedded in Matrigel. However, this method showed low tumourigenic efficiency and was not considered a good model [141]. More recently, another chemically induced RC model was developed. In this case, a compound that methylates specific regions in the DNA, N-methyl-Nʼ-nitro-N-nitrosoguanidine (MNNG), was used in C57BL/6 mice. This methodology induced perianal inflammation, mucosal lesions, and tumour development, with high inter-mouse variability and no evidence of metastases. Of note, in this report, disease progression was monitored via low-endoscopy, highlighting a novel methodology to advance murine longitudinal studies [142]. Minicozzi et al. proposed that trans-anal RC cell injection (TARCI) as the most effective and less invasive technique for establishing an RC animal model. Accordingly, the authors used TARCI to inject human HT29 colon cancer cells directly into the submucosal layer of athymic nude mice rectums, resulting in the consistent development of primary tumours and lymph node metastases in all animals, highly mimicking human disease [143]. However, this method relies on human cancer cells and requires immunodeficient mouse strains, precluding the study of interactions with an intact immune system. Despite their central role in RC research, existing animal models still fall short of providing a fully reliable platform to study RC biology and therapeutic responses. On the one hand, models based on human cells require immunodeficient hosts, limiting the investigation of tumour-immune system interactions. In fact, the lack of well-characterised and established murine rectal cancer-specific cell lines, in contrast to the widely used murine colon cancer cell lines MC38 and CT26, hinders the development of truly orthotopic and immunocompetent rectal cancer models. Nevertheless, a genetically engineered murine organoid orthotopic model has been reported and used in the context of image-guided radiation therapy, opening the window in this field for future developments [81, 144].

## Translational implications for clinical practice

The research on RC biomarkers reviewed in this manuscript provides mechanistic insight into how deregulated pathways, including DNA damage repair, cell cycle regulation, hypoxia, apoptosis, stromal-tumour microenvironment interactions, and cellular metabolism, may influence the biological effects of radiotherapy and, ultimately, treatment response (Fig. 1). However, a key unresolved question remains about how knowledge of deregulated molecular pathways can be translated into clinical decision-making for patients undergoing radiotherapy treatment.

To address this question, the following sub-sections integrate relevant radioresistance biomarkers with current management of LARC, encompassing pre-treatment risk stratification and regimen choice, as well as post-treatment decisions regarding surgery, adjuvant therapy, and surveillance.

### Pre-treatment: patient selection and risk stratification

DNA damage repair, apoptosis, and hypoxic biomarkers (e.g., PRDM15, Ku70/Ku80, NONO–RPLP0, TTI1, XIAP, survivin/BIRC5, SGK1, REG4/NEIL2, HIF‑1α) together with metabolic adaptations (OXPHOS‑ and lipid‑rewiring signatures like COASY, PITPNC1, ABCC4, Fig. 1) could be used in biopsy screenings as a pre-treatment strategy for risk stratification. Patients with high-risk biomarker profiles, such as those previously stated, could be redirected to more appropriate regimens, early surgery, and, more importantly, inclusion in clinical trials testing radiosensitising agents.

Nevertheless, only recently has RC biomarker research reached clinical trials, but still with limited success. In 2016, a clinical study trial (NCT02584400), led by Maastricht Radiation Oncology used the [18F]HX4 hypoxic marker to select RC patients with hypoxic tumours who would benefit from anti-hypoxic treatments (Table 1). Nevertheless, this clinical trial was terminated in 2017 due to constraints on patient enrolment. Similarly, a clinical study (NCT02157246) conducted by Oxford University, which employed 18F-fluoromisonidazole (F-MISO), was designed to identify hypoxic tumours and to correlate them with radiotherapy treatment and patient response. However, fundamental difficulties in data interpretation limited clinical applicability [145]. A phase I clinical trial (NCT04406857) led by the National Cancer Institute in the USA evaluated the optimal dose and side effects of Ropidoxuridine, a DNA strand break agent, as radiotherapy sensitising for stage II-III RC patientsʼ treatment. Nevertheless, this study terminated due to low patient enrolment [146]. Additionally, the ACO/ARO/AIO-21 phase I clinical trial (NCT04942626) evaluated capecitabine-based chemoradiotherapy in combination with the IL-1 receptor antagonist anakinra in RC patients, showing a manageable safety profile and supporting further clinical investigation of this anti-inflammatory approach together with standard nCTR regimens [147]. A very interesting, and still active, clinical study (NCT06831669), led by Chang Gung Memorial Hospital, aims to evaluate the metabolomic profile by chromatography-mass spectrometry (LC–MS) of blood, urine, faecal, and tissue samples of LARC patients before, during and after TNT to predict treatment response. The main objective is to select patients most likely to benefit from TNT, while minimising the risk of treatment-associated complications. Another clinical trial, conducted at the University of Kansas Medical Centre (NCT03874559), aims to assess exosome expression levels in blood samples from LARC patients, comparing them before and after nCRT and correlating results with patient pathologic response. Similarly, IRCCS Azienda Ospedaliero-Universitaria di Bologna (NCT06730035) aims to determine, by blood analysis, whether circulating extracellular vesicles and microRNAs can predict clinical and pathological responses to radiotherapy in LARC patients. Table 1 summarises the RC clinical trials mentioned above and explores the combination of biomarker research to potentiate radiotherapy response in LARC patients.

**Table 1:** Clinical trials exploring the combination of biomarker research to potentiate radiotherapy response in LARC patients. (accessed information from https://www.clinicaltrials.gov/ on February 2026)

**Status**	Terminated	Completed	Terminated	Recruiting	Recruiting	Active, not recruiting	Recruiting	Completed	Completed
Location	Maastricht Radiation Oncology, The Netherlands	University of Oxford, Great Britain	National Cancer Institute, USA	Chang Gung Memorial Hospital, Taiwan	University of Kansas Medical Center, USA	IRCCS Azienda Ospedallero Universitaria, Bologna, Italy	Washington University School of Medicine, USA	AHS Cancer Control, Alberta, Canada	University Hospital Goethe University Frankfurt, Germany
ID number	NCT02584400	NCT02157246	NCT04406857	NCT06831669	NCT03874559	NCT06730035	NCT03516708	NCT02569645	NCT04942626
Sample type	Injection administration	Biopsy, blood	Drug administration	Blood, urine, faeces, tumor	Blood, serum	Peripheral blood	Drug administration	Drug administration	Drug administration
Biomarker	Hypoxia	Hypoxia	DNA strand-break agent	Metabolic activity	Exosomes	Extracellular vesicles/miRNAs	IDO1, tryptophan catabolism	HMG-CoA reductase, Cholestrol metabolism	Immune Modulation
Number of patients	1	14	1	Recruiting, estimated 250	30	30	57	48	12
Clinical phase	II	-	I	-	-	-	I/II	II	I
Treatment	Injection with Hypoxia tracer [18F]HX4 and PET imaging	Rectal cancer sample, F-MISO PET, pCT, functional MRI	Neoadjuvant Ropidoxuridine + capecitabine + SCRT followed by standard of care surgery (8–12 weeks) after treatment	Prospective LC–MS analysis in RC patients DNA samples	Prospective exosomal biomarker characterization	Prospective Extracellular vesicles, miRNAs characterization	Dose Escalation Cohort (Phase I): Epacadostat + SCRT + CT + Surgery Treatment Cohort (Phase Ii): Epacadostat + SCRT + CT + Surgery	Rosuvastatin orally starting 2 weeks prior RT and stopped 4 weeks after completion RT	Capecitabine-based CT in combination with IL-1 R Antagonist Anakinra osuvastatorally starting 2 weeks prior RT and stopped 4 weeks after completion RT

Of note, RC patients with low-risk biomarker profiles should use, whenever possible, standard nCRT to enhance organ preservation. Additionally, it would be important to include the analysis of immune and stemness markers (CD8^+^ and CD4^+^/FOXP3^+^TIL density, complement activation, BMI1/OCT4 and EMT‑related CSC signatures, Fig. 1) before patient treatment to predict the probability of pCR, assisting in patient selection for a ʼwatch‑and‑waitʼ approach and avoiding the commonly performed total mesorectal excision.

### Post-treatment

One of the main issues in RC radioresistance is the development of therapy resistance. In patients with a near-complete pathological response and residual high-risk biomarker patterns (e.g., persistent XIAP/survivin, strong CSC/EMT signatures, high CXCL12-FAP iCAF activity; Fig. 1) in post-treatment resections, a surgical approach might be advantageous over watch-and-wait. More interestingly, RC patients with decreased tumour regression after nCRT could benefit from analysis of several biomarkers, including DDR/apoptosis, PI3K–AKT–mTOR activation, metabolic rewiring, and TME composition (Fig. 1), to assist in adjuvant systemic therapy decisions rather than relying on uniform chemotherapy regimens. A Phase I/II clinical trial (NCT03516708) is currently investigating whether Epacadostat, an inhibitor of the enzyme indoleamine 2,3-dioxygenase 1 (IDO1), can increase tryptophan availability and, consequently, increase T cell and NK cell activation while decreasing Treg infiltration (Table 1) [148, 149]. This trial also aims to evaluate whether Epacadostat, in combination with SCRT (5 Gy/5 days) and chemotherapy (capecitabine and oxaliplatin), further reduces tumour burden before surgery. Another clinical study from AHS Cancer Control, Alberta, Canada (NCT02569645) evaluated the addition of Rosuvastatin, an HMG-CoA reductase inhibitor, a crucial enzyme in cholesterol production [150], to improve pCR in RC patients treated with nCRT. The authors have shown that adding rosuvastatin to nCRT resulted in a high complete and near-complete response rate with an acceptable toxicity profile [151].

## Conclusions and future perspectives

Since the first RC cells were established in 1972, radioresistance research has remained a major obstacle in achieving optimal therapeutic outcomes. Despite decades of research, its multifactorial nature continues to challenge the identification of all the molecular and cellular mechanisms involved. RC radioresistance arises from a complex interplay of processes, including modulation of the TME, through immune cell interactions, fibroblast activation, and hypoxia, that promote survival under therapeutic pressure while enhancing invasive and metastatic potential. It also accounts for intrinsic tumour cell responses, cell cycle arrest, DNA damage repair, and the induction of stemness, as well as patients overall status and tumour characteristics. Moreover, accumulating evidence suggests that metabolic reprogramming enables RC cells to adapt to the oxidative and energetic stress imposed by radiotherapy. Therefore, we propose that this metabolic adaptability, which counteracts ionising radiation-induced cell death, should be recognised as the “seventh R” of radiobiology. Collectively, these dynamic alterations within the tumour ecosystem underscore the complexity of RC radioresistance and highlight interesting candidates for clinical studies.

The emergence of 3D culture systems, such as spheroids and PDOs, together with animal models, has revolutionised our ability to study tumour responses under conditions that better mimic *in vivo* architecture and microenvironmental interactions. These systems have already provided crucial insights into the mechanisms by which RC cells and their surrounding microenvironment resist ionising radiation, not only advancing the discovery of biomarkers for basic research. However, they can serve as pre-clinical companions to validate whether candidate biomarkers truly benefit from radiosensitising agents or immune‑targeted combinations, as well as for fraction-dose personalisation. Furthermore, how does this influence practice? By positioning the pre-clinical models as the vehicle for implementing biomarker‑guided radiotherapy. Some of the reviewed biomarkers, namely PRDM15, XIAP, and survivin, as well as the TME composition, before and after LARC treatment, have already shown prognostic and predictive value. Nevertheless, prospective validation in nCRT/TNT trials would be crucial. Other biomarkers, especially focusing on metabolic alterations in cholesterol and tryptophan metabolism, have already shown, prospectively, a decrease in tumour burden as radiosensitisation agents.

Nevertheless, several gaps continue to hinder biomarker research. Most DDR, cell death, TME, and metabolic markers are derived from heterogeneous preclinical systems, including 2D cultures, 3D spheroids, PDOs, and animal models, that differ substantially in culture conditions, ionising radiation schemes, and endpoint readouts. In addition, clinical evidence often relies on small, retrospective cohorts with variability in radiotherapy dose and fractionation regimens.

Additionally, most studies do not integrate the 7Rs of radioresistance, and evidence suggests that all these aspects may occur within the tumour. Integrative multi-omics, including transcriptomics, proteomics, and metabolomics, from 3D models and pre- and post-treatment biopsies, can provide a mechanistic signature of radioresistance. Additionally, while most studies are “static” with single time point before or after therapy, the sequence of events leading to resistance (e.g., early metabolic rewiring and immune system remodulation) is unclear. Therefore, the need for longitudinal sampling (serial biopsies, circulating ncRNAs/metabolites/proteins, serial PDO generation) and time‑resolved modelling of resistance trajectories under clinically relevant radiotherapy schemes would be of relevance.

Furthermore, more recently, the development of particle therapies, such as proton and carbon ion therapy, magnetic resonance–guided linear accelerators, and ultra-high dose-rate approaches (FLASH), has further refined dose conformity and biological selectivity. Indeed, although photon beams are the most commonly used form of external radiation in cancer therapy, proton therapy offers a dosimetric advantage. Unlike photon radiation, the beam does not continue depositing significant energy beyond the tumour. Instead, the majority of the dose is released in the final millimetres of the protonʼs path, a phenomenon known as the Bragg peak, where the dose is concentrated mainly within the targeted tumour region, reducing damage to adjacent normal structures [152]. Additionally, FLASH radiotherapy has emerged as an innovative approach to cancer treatment, delivering ultra-high doses of radiation in a fraction of a second (> 40 Gy/s), being significantly faster than traditional radiotherapy (0.1 Gy/s) [153]. This technique is designed to target tumours while sparing surrounding healthy tissues. The underlying principle is known as the “FLASH effect”, where normal tissues exhibit less damage when exposed to such fast doses compared to conventional radiotherapy methods [154]. Interestingly, FLASH radiotherapy could revolutionise cancer radiobiology, making treatments not only faster but also safer and more effective. Clinical trials are ongoing to establish safety, efficacy, and long-term outcomes in patients. The first clinical study of FLASH radiotherapy was conducted in 2018 [155]. A 75-year-old patient diagnosed with T-cell skin lymphoma received a total dose of 15 Gy delivered in 10 pulses, each pulse lasting for 1 µs. The tumour response was fast, complete, and durable with a follow-up time of 5 months. Only grade 1 dermatitis and oedema were observed after this initial treatment [155]. Additionally, in 2021, 10 patients with painful bone metastases were given 8 Gy of radiation in a single fraction, delivered at ≥ 40 Gy per second (NCT04592887). Seven patients experienced complete or partial pain relief. In 6 of the 12 sites (50%), patients reported a complete response [156]. Side effects from treatment were mild and consistent with conventional radiotherapy. Hence, these new innovative radiotherapy modalities offer a way to minimise side effects while maintaining, or even improving, the efficacy of cancer treatment, being a way of possibly reducing rectal cancer radioresistance [156].

Ultimately, the integration of these advanced experimental systems with translational prospective studies will be crucial for overcoming the enduring challenge of radioresistance in RC, reducing the risk of relapse and metastatic progression.

## Data Availability

No datasets were generated or analysed during the current study. not applicable.
